# Effects of an intergenerational service-learning program on prosocial behaviors and perception of physical education in primary education students

**DOI:** 10.3389/fpsyg.2025.1650084

**Published:** 2025-09-24

**Authors:** Encarnación E. Ruiz-Montero, Horacio Sánchez-Trigo, Oscar Chiva-Bartoll, Pedro Jesús Ruiz-Montero

**Affiliations:** ^1^Department of Physical Education and Sport, University of Granada, Granada, Spain; ^2^TEPAS Research Group HUM-1080, Department of Physical Education and Sport, Faculty of Sport Science, University of Granada, Granada, Spain; ^3^Department of Physical Education and Sport, University of Sevilla, Seville, Spain; ^4^Department of Education and Specific Didactics, Faculty of Humanities and Social Sciences, University Jaume I of Castellón, Castellón, Spain; ^5^Department of Physical Education and Sport, Faculty of Sport Science, University of Granada, Granada, Spain

**Keywords:** service-learning, prosocial behaviors, intergenerational education, social inclusion, values education

## Abstract

**Introduction:**

The Service-Learning (SL) promotes the acquisition of curricular competencies through enriching experiences that respond to real social needs, within a framework of critical reflection and ethical commitment. Through an intergenerational SL program, this article addresses the global phenomenon of population aging. Preventing ageism as a potential discriminatory behavior should be the foundation for building positive prosocial behaviors from childhood. In addition, Physical Education (PE) can be an effective tool to promote social inclusion.

**Methods:**

A total of 106 students, divided into two methodology groups (Traditional vs. Service-Learning; age = 10.49 ± 0.50), participated in a 10-week intervention with older adults in a day center, using physical activities as the main tool. The study incorporated standardized questionnaires assessing prosocial behaviors (PB) and students’ self-perception of PE lessons.

**Findings:**

The results show significant improvements in most PB dimensions (Empathy, Respect, Sociability, and Leadership; all, *p* < 0.05) and in self-perception of the PE subject in the SL group (*p* < 0.05), in contrast to the TM group, which showed no improvements.

**Discussion:**

In general, an experiential methodology with an inclusive focus such as SL fosters civic and respectful behaviors in adolescents, especially when they engage with socially disadvantaged groups. Furthermore, the role of PE is crucial, as it enables direct and progressive interaction with the target population, in this case, older adults.

## 1 Introduction

Service-Learning (SL) has emerged in recent decades as one of the most effective experiential approaches for integrating academic learning with social action. It is defined as a pedagogical model that combines teaching processes with community service, allowing students to learn while actively contributing to the improvement of their social environment (Drewery and Lollar, 2024). This methodology promotes the acquisition of curricular competencies through meaningful experiences that address real social needs within a framework of critical reflection and ethical commitment.

From a socio-educational perspective, SL fosters fundamental values such as empathy, responsibility, respect, and solidarity, aligning with the goals of holistic education and active citizenship promoted by educational systems (Coelho and Menezes, 2021). In this regard, SL transcends the mere transmission of content, becoming a transformative learning experience for both students and the communities they engage with.

Despite the increasing recognition of SL as an educational tool, its research in Primary Education remains limited, especially in cases involving structured interventions outside the school environment and with vulnerable populations. Most studies on SL have been conducted in secondary or higher education, where students typically demonstrate greater autonomy and maturity. However, various authors argue that upper-primary students already possess the cognitive, emotional, and social development necessary to actively and responsibly engage in community service projects (Calvo Varela et al., 2019; Zorrilla-Silvestre et al., 2019).

Intergenerational SL experiences in educational contexts represent a valuable pedagogical strategy by promoting contact between children and older adults. Interaction between different generations facilitates the exchange of knowledge, recognition of diversity, and deconstruction of age-related stereotypes (Wang, 2021). Similarly, Gualano et al. (2018), in a systematic review, concluded that intergenerational programs lead to significant improvements in children’s perceptions of aging, increasing empathy, respect, and sensitivity toward older adults.

In addition to the benefits observed in students, numerous studies have emphasized the positive effects of SL on older adults participating in intergenerational programs. Research has shown that such interventions can lead to improved mood, increased social interaction, and enhanced feelings of usefulness and self-worth among older participants (Gualano et al., 2018). Specifically, when children or adolescents are involved in service activities with dependent older adults, the elderly often experience a reduction in perceived loneliness and a sense of emotional connection, which can contribute to their overall well-being (Skropeta et al., 2014). These findings underscore the reciprocal nature of SL, which not only supports students’ learning and development but also serves as a valuable tool for promoting the social inclusion and quality of life of older adults.

In the Spanish context, where the present study is situated, a notable example is the “Compartir la infancia” (Sharing Childhood) program, which has shown that participating children develop more positive attitudes toward older adults and toward individuals with cognitive or functional diversity (Vives et al., 2021). While most of these studies focus on attitudinal changes, others such as Ruiz-Montero et al. (2024) have explored the development of specific competencies, highlighting that intergenerational SL in Physical Education (PE) not only fosters empathy but also promotes prosocial leadership among Primary students.

In cases where such programs have not been implemented in Primary Education, studies involving adolescents close to the age range of the current sample (10–11 years) point to similar benefits, particularly regarding responsibility, inclusion, and motivation for learning (Gregorová et al., 2024).

PE is one of the most highly valued subjects among primary school students. Various studies have documented how this subject is perceived not only as a space for play and enjoyment, but also as meaningful and useful for everyday life (Adank et al., 2024; Moreno-Murcia et al., 2009; Moreno-Murcia et al., 2013). Students associate PE with positive emotions, opportunities for socialization, and the development of both physical and personal skills. This perception is further reinforced when the content addressed transcends the school setting and has a direct impact on the community.

In SL contexts, the value attributed to PE increases, as students experience how their learning has real-world impact. For instance, Ruiz-Montero et al. (2024) demonstrated that the perception of PE’s importance even acts as a mediating variable in the development of prosocial behaviors. In other words, when students value PE, they are more motivated to engage, collaborate, and lead service-oriented activities, such as those implemented in SL programs. This finding confirms that PE is a privileged channel for fostering civic engagement and social awareness from an early age.

One of the main contributions of SL is its ability to foster prosocial behaviors (PB), understood as voluntary actions aimed at benefiting others (Chiva-Bartoll et al., 2020; Pallás et al., 2011). PB is expressed through four interrelated dimensions: empathy, respect, sociability, and prosocial leadership. Empathy refers to the ability to recognize and respond to others’ emotions and needs; respect involves adopting inclusive attitudes and behavior toward others, especially older adults in this context; sociability encompasses interpersonal communication, cooperation, and participation in group settings; and prosocial leadership reflects the capacity to guide, support, and motivate others through collaborative and ethically grounded actions. These dimensions can be actively developed through specific practices within SL in PE. For instance, empathy is strengthened through direct interaction with vulnerable populations—such as dependent older adults—enabling students to understand emotions, recognize limitations, and respond compassionately (Gualano et al., 2018; Chiva-Bartoll et al., 2020). Respect is reflected in students’ behavior, such as adapting tasks to older adults’ needs, using appropriate language, and accepting their pace (Ruiz-Montero et al., 2020). Solidarity emerges as students apply their knowledge to benefit others without expecting material rewards, fostering shared responsibility and social commitment (Bunsi et al., 2024; Guerra-Marmolejo et al., 2024). Finally, SL in PE creates opportunities for prosocial leadership, as students take initiative, guide activities, and promote inclusive participation grounded in service values (Badenes-Ribera et al., 2023; Caprara et al., 2006; Ruiz-Montero et al., 2024). Taken together, these dimensions shape a prosocial profile that extends beyond the classroom and into the broader community. SL, particularly when integrated with PE, generates authentic learning contexts where these competencies are practiced and reinforced.

On the other hand, SL programs focused on older adults’ health might be a useful step in promoting positive attitudes toward working with older adults on students (Kimzey et al., 2016; Lokon et al., 2017), especially in PE lessons. In addition, several authors agree that the SL is an optimal way to promote intergenerational social cohesion between young and older adults (Bunting and Lax, 2019; Heuer et al., 2019; Ruiz-Montero et al., 2020).

The present study aims to compare the effects of traditional methodology (TM) and SL on two core variables: the perceived importance of PE and the development of PB in primary school students. The perceived importance of PE refers to the value students attribute to the subject in relation to their personal growth, social interactions, and ability to contribute meaningfully to the well-being of others. PB is examined through the four previously described dimensions: empathy, respect, sociability, and prosocial leadership.

By analyzing changes in students’ perceptions and behaviors following the implementation of an intergenerational SL intervention with dependent older adults, the study seeks to provide empirical evidence of the educational value of this methodology. Ultimately, it highlights the potential of PE not only as a site for physical development but also as a transformative space for fostering civic values and social inclusion from an early age.

## 2 Methods

### 2.1 Participants

The study included a total of 106 students, ultimately divided into a Traditional Methodology (TM) group (*n* = 38; 24 female and 14 male students) and a SL group (*n* = 35; 21 female and 14 male students), with an average age of 10.11 ± 0.31 years for TM group and 10.91 ± 0.28 on the SL experience participants.

The participation of students in the experimental group in the SL project was not voluntary, as it was part of a school project. Before the intervention outside the school, students were informed regarding their tasks based on older adults needs: Promote physical activity by developing interventions similar as those implemented by physical education instructors. The students also received a lecture on intergenerational education addressing key issues regarding aging and inclusion developed by an expert on intergenerational education.

On the other hand, the control group’s participation in PE sessions was based on games, exercises and physical fitness activities, as was the experimental group, but adapted to the context of a school playground-gym. In addition, both the experimental group and the control group attended a talk given by a professional in the field of Intergenerational Education and physical activity.

The research design was a quasi-experimental quantitative study, conducted as a longitudinal design over two-thirds of the academic year. The four classes (two fifth graders and two sixth graders) were assigned non-randomized so that the fifth graders constituted the control group (TM) and the sixth graders the experimental group (SL). The control group was instructed using a TM approach, which involved lecture-based teaching, standardized evaluation, and limited emphasis on active participation. In contrast, the SL group participated in an intervention based on an experiential methodology.

**Figure 1:**
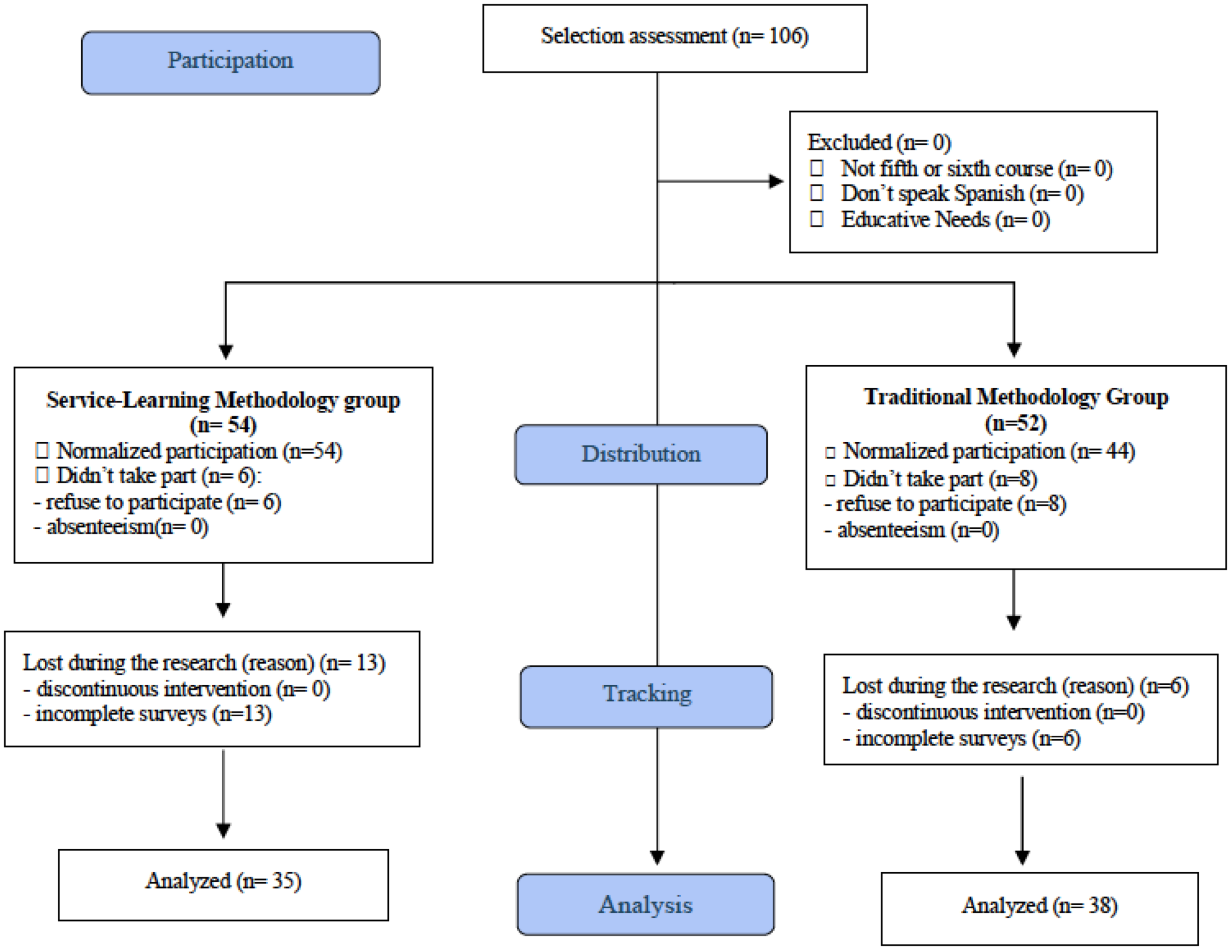
Flowchart of participants (adapted from CONSORT flow diagram).

The following exclusion criteria were applied: (i) students not enrolled in the fifth or sixth year of Spanish primary education, (ii) students who did not speak Spanish or had significant language difficulties, and (iii) students requiring major curricular adaptations due to classification as having Special Educational Needs. A visual representation of the participant flow is provided in Figure 1.

### 2.2 Measures

To collect sociodemographic information, a tailored questionnaire was individually administered with the supervision of responsible of the present study. The questionnaire was administered during school hours in the classroom setting. It included the following variables: (a) gender (male or female), (b) instructional methodology (TM or SL), (c) average age and standard deviation by group, (d) items from the Physical Education Importance Questionnaire (PEIQ), and (e) items and dimensions from the PB Questionnaire.

The following standardized instruments were used:

- *PEIQ* (Moreno-Murcia et al., 2009): This instrument includes three items assessing students’ perceptions of the significance and practical value of PE. The items are: (a) “I believe PE classes are important,” (b) “Compared to other subjects, PE is one of the most valuable,” and (c) “The knowledge gained in PE will be beneficial in my daily life.” Responses are recorded using a 4-point Likert scale ranging from 1 (“Strongly disagree”) to 4 (“Strongly agree”). The reliability coefficient of the PEIQ is 0.75, indicating satisfactory internal consistency.
- *PB Questionnaire* (Pallás et al., 2011): This questionnaire consists of 58 items across four dimensions: Empathy (17 items), Respect (15 items), Sociability (15 items), and Leadership (10 items). Each item is rated on a 4-point Likert scale from 1 (“Never”) to 4 (“Always”). Designed for children and adolescents under the age of 17, the questionnaire demonstrates strong psychometric properties, with internal consistency values ranging from 0.76 to 0.87 and test–retest reliability scores between 0.65 and 0.71 (Pallás et al., 2011).

### 2.3 Design and procedure

The study was conducted in accordance with the ethical principles of the Declaration of Helsinki and received approval from the Ethics Committee of the University of Granada, Spain (ref. 3516/CEIH/2023), ensuring full compliance with research responsibilities. Furthermore, the study was part of a regionally funded educational innovation project titled “*Education in Values and Intergenerational Education: A Pillar of Connection for Students Between Who We Were, Who We Are, and Who We Want to Be*.”

Regarding the selection and contact process, the participants were enrolled at a public primary school in the province of Málaga, Spain. Since the participants were under 18 years of age; families were provided with detailed information about the research process in an initial meeting held at the beginning of the school year. Just before the implementation of the project, an information document was sent along with informed consent forms authorizing their children’s participation (Wendler, 2006). The invitation to participate was sent through the official Family Education Platform (Ipasen) of the Andalusian Regional Government, the region where the study was conducted. These families gave their consent or not, and these decisions were recorded. The questionnaires gathered data on the study variables as well as demographic information and were administered 1 week before and 1 week after the intervention. The pre-test and post-test questionnaires were administered individually to each student, and any questions that arose during the completion process were clarified.

The school year in primary education in Spain lasts 37 teaching weeks. The SL intervention program was carried out over a period of 10 weeks, with two physical education sessions per week, each lasting between 45 and 50 min, during the 2022/2023 academic year. The educational content focused on students’ perceptions of PE and the development of prosocial competencies. These contents were aligned with the official PE curriculum and included three learning situations: “Discovering Well-being” (physical capacities: strength and endurance), “Flexible Body Challenge” (physical capacity: flexibility), and “Cooperative Challenges” (physical capacity: endurance). The PE teacher was responsible for promoting her students’ active engagement in class, setting goals, and always integrating them into the group. The reason was to foster positive motivation in the students, increasing their perception of the relevance and benefits of PE in their daily lives. Therefore, participation in the SL was active and exciting for all students, including those who, for various reasons, did not complete the questionnaires.

This approach contrasts with traditional methods; while the TM group carried out activities within the school setting, the SL group engaged in a service-learning intervention at a local day center for older adults (aged 55 and above). The intervention was organized into three main phases. First, the participants of the SL group were introduced to the context and objectives of the intervention through group dynamics, viewing of prior SL experiences, and awareness-raising activities aimed at understanding the realities of socially vulnerable populations. At this stage, participants also completed the pre-intervention questionnaires.

In the second phase, SL sessions were carried out at the day center, where students applied the knowledge and skills they had prepared in a real-world environment. The SL sessions, conducted by the students with the older adult group, were based on games, exercises, and physical activities (flexibility, strength, agility, and endurance), with simultaneous sessions constantly supervised by the teacher in charge of the student group at the day center. Physical therapists and psychologists from the day center also assisted and supervised the experience to ensure the smooth running of the SL sessions; for example, in a strength session, joint mobility exercises and a slight increase in heart rate were performed during the warm-up, followed by a fun self-loading exercise in pairs of equals (student with student, older adult with older adult): they placed their hands on their face and their partner removed their hands; they brought their hands together and their partner separated them…

Finally, in the third phase, post-intervention questionnaires were administered to collect and systematize the outcomes of the experience. The procedural phase for administering the instrument to students, based on pre- and post-test questionnaires, was organized individually with each student to ensure equal conditions for all. The classroom context provided a suitable environment for its implementation. The individualization of the process was intended to ensure a correct understanding of both the instructions and the content of the questions.

This methodological approach aligns with prior research highlighting the value of SL as a pedagogical tool for promoting inclusion, social engagement, and the development of personal and social competencies in educational settings (Calvo Varela et al., 2019).

### 2.4 Statistical analysis

Sociodemographic variables are presented as mean ± standard deviation (age) or as frequencies and percentages for categorical variables (e.g., PE Importance items and Prosocial Behaviors dimensions). Differences between pre- and post-intervention scores for both groups (TM and SL) were analyzed using the Wilcoxon signed-rank test for non-parametric paired data on the PE Importance Scale items and the Prosocial Behaviors dimensions. The magnitude of change between time points was assessed using effect size (η^2^), following Cohen’s guidelines (Cohen, 1992), with thresholds interpreted as small (0.2 < *d* < 0.5), medium (0.5 < *d* < 0.8), and large (*d* ≥ 0.8).

Additionally, we included the change scores (post – pre) from both the PE Importance Scale and the Prosocial Behaviors dimensions as dependent variables in linear regression models, with group (TM vs. SL) as the independent variable. Two models were estimated: Model I was unadjusted; Model II was adjusted for age, gender (female/male/other), and group (TM or SL), as previous studies have shown age to be positively associated with PE Importance and Prosocial Behaviors, and female students to exhibit higher PB and lower PE Importance (Ruiz-Montero et al., 2024).

All statistical analyses were performed using IBM SPSS Statistics for Windows, Version 25.0 (IBM Corp., Armonk, NY, United States). Statistical significance was set at *p* < 0.05.

## 3 Results

Table 1 presents the pre–post differences observed after the 10-week intervention for the three items of the PEIQ and the dimensions of PB, namely Empathy, Respect, Sociability, and Leadership. Significant improvements were observed in the SL intervention group (all, *p* < 0.05). Specifically, among primary school students in the SL group, Item 2 of the PEIQ as well as the PB dimensions of Empathy, Respect, and Sociability showed statistically significant changes following the intervention (all, *p* < 0.05).

**Table 1:** Per-protocol analyses showing the post-pre differences on the PE importance and prosocial behaviors after 10-week intervention program with the intervention group (TM and SLM) on primary school students.

	Service-learning methodology (*n* = 35)				Traditional methodology (*n* = 38)			
	Post	Pre	*p*-value	Cohen’s d	Post	Pre	*p*-value	Cohen’s d
PE importance								
1. I think it is important to receive PE classes	3.62 (0.68)	3.65 (0.53)	0.739	0.048	3.92 (0.27)	3.86 (0.34)	0.414	0.195
2. Compared with the rest of the subjects, I think that PE is one of the most important	3.14 (0.80)	2.77 (0.84)	0.040	0.451	3.18 (0.72)	3.07 (0.58)	0.400	0.168
3. I think the things I learn in PE will be useful in my life	3.17 (0.78)	3.08 (0.85)	0.624	0.110	3.10 (0.64)	3.21 (0.77)	0.518	0.155
Prosocial behaviors								
Empathy	3.30 (0.34)	3.05 (0.44)	0.017	0.635	3.07 (0.44)	3.39 (1.29)	0.349	0.332
Respect	3.06 (0.28)	2.84 (0.35)	0.011	0.694	2.92 (0.29)	2.85 (0.31)	0.762	0.233
Sociability	3.12 (0.25)	2.97 (0.34)	0.032	0.502	3.06 (0.28)	2.98 (0.32)	0.267	0.266
Leadership	3.07 (0.42)	2.78 (0.54)	0.059	0.599	3.07 (0.38)	2.96 (0.39)	0.536	0.285

Table 2 displays the per-protocol analysis comparing changes between pre- and post-intervention values for the TM and SL groups. In the unadjusted model (Model I), no significant differences were detected. However, after adjusting for age, gender, and total group (Model II), results revealed a marginal increase in Item 2 of the PEIQ across the SL group by 0.4% whereas the TM group increased it by 0.1% (95% CI: −0.015 to 1.388; *p* = 0.055). Additionally, gender was found to significantly influence the Leadership dimension of PB, showing an increase of 0.3% for SL group and 0.1% for TM group (95% CI: 0.012 to 0.596; *p* = 0.041).

**Table 2:** Per-protocol analyses showing the associations of changes on the PE importance and prosocial behaviors after 10-week intervention program with the intervention group (TM and SLM) on primary school students.

Questionnaire			Model I		Model II	
	Changes within SLM group, post-pre (*n* = 35)	Changes within TM group, post-pre (*n* = 38)	Between-group difference^a^ (95% CI)	*p*-value	Between-group difference^a^ (95% CI)	*p*-value
PE importance						
1. I think it is important to receive PE classes	−0.028 (0.513)	0.526 (0.399)	0.024 (−0.295, 0.133)	0.451	TG = 0.348 (−0.540, 0.192)	0.347
					G = 0.177 (−0.130, 0.307)	0.421
					A = 0.239 (−0.247, 0.486)	0.517
2. Compared with the rest of the subjects, I think that PE is one of the most important	0.371 (1.002)	0.105 (0.763)	0.266 (−0.148, 0.680)	0.204	TG = 1.373 (−0.015, 1.388)	0.055
					G = 0.191 (−0.323, 0.514)	0.649
					A = 1.404 (1.216, 0.188)	0.149
3. I think the things I learn in PE will be useful in my life	0.085 (1.010)	−0.105 (1.007)	0.019 (−0.280, 0.662)	0.422	TG = 0.191 (−0.707, 0.898)	0.814
					G = 0.637 (−0.160, 0.797)	0.188
					A = 0.274 (−0.666, 0.940)	0.735
Prosocial behaviors						
Empathy	0.245 (0.572)	0.495 (0.510)	0.195 (−0.057, 0.449)	0.127	TG = 0.129 (−0.368, 0.497)	0.768
					G = 0.192 (−0.162, 0.354)	0.461
					A = 0.337 (−0.264, 0.601)	0.440
Respect	0.213 (0.464)	0.658 (0.377)	0.125 (−0.049, 0.344)	0.140	TG = 0.262 (−0.207, 0.469)	0.442
					G = 0.162 (−0.121, 0.283)	0.426
					A = 0.050 (−0.313, 0.363)	0.884
Sociability	0.149 (0.454)	0.081 (0.414)	0.067 (−0.135, 0.270)	0.508	TG = 0.392 (−0.151, 0.543)	0.264
					G = 0.068 (−0.173, 0.241)	0.744
					A = 0.313 (−0.504, 0.191)	0.371
Leadership	0.288 (0.741)	0.110 (0.501)	0.178 (−0.115, 0.471)	0.230	TG = 0.563 (−0.208, 0.771)	0.255
					G = 0.584 (0.012, 0.596)	0.041
					A = 0.221 (−0.600, 0.379)	0.655

TM, Traditional Methodology; SLM, Service-Learning Methodology; CI, Confidence interval; A, Age; G, Gender; TG, Total group.

^a^Values represent the non-standardized mean difference between groups, expressed as mean (standard deviation). Model I is unadjusted. Model II is adjusted for age and gender (female/male/other). The means indicate the difference between post- and pre-intervention scores (i.e., from baseline to the end of the 10-week SLM intervention). Negative values indicate a decrease in the post-intervention assessment compared to baseline.

## 4 Discussion

The main objective of this study was to compare the effects of TM and SL on final-year primary students’ perceptions of PE and their prosocial competencies. Specifically, we analyzed how each teaching approach (TM vs. SL) influenced students’ engagement and the value they placed on PE classes. Additionally, we evaluated changes in prosocial behavior among students following the implementation of the SL. Based on the findings (Table 2), improvements were observed in several variables within the experimental group after the service-learning intervention. These improvements were not seen in the control group, suggesting that the intervention had a positive impact on students who participated in the SL program.

Regarding students’ perception of the importance of PE, the experimental group showed a significant improvement in how they ranked PE compared to other school subjects. In other words, the SL intervention led to a shift in perception, with students expressing that PE was among the most valuable subjects in the curriculum. These results are consistent with previous research showing that SL can positively influence students’ attitudes toward the subjects in which it is applied (Li et al., 2019; Zucchero and Gibson, 2019). This effect, considering how important is supporting autonomy for the perception of the PE subject (Aguado et al., 2016), may be partly attributed to the experiential nature of SL, which demands a high level of engagement and autonomy (Chiva-Bartoll and Fernández-Río, 2022; Lee and Perdana, 2023; Rodríguez-Zurita et al., 2025).

However, no significant changes were found in the remaining PEIQ items. Thus, while students already considered PE an important subject prior to the intervention, participating in the SL experience further reinforced their perception of PE’s value relative to other subjects. These findings support the assertions of Campos-Rius et al. (2020), who note that SL provides renewed meaning to PE learning experiences. Again, the level of student involvement and commitment required by SL appears to be a distinguishing factor when compared to other pedagogical models (Furco, 2010).

In terms of prosocial variables, improvements were noted in Empathy, Respect, and Sociability within the experimental group. These improvements were not observed in the TM group. The literature broadly supports the idea that SL enhances prosocial development (Billig, 2000; Chan et al., 2021) by fostering a deeper understanding of civic values. In this sense, previous research shows that applying SL in PE enhances civic values and attitudes (Calvo Varela et al., 2019; Ruiz-Montero et al., 2023). In the context of primary education, Castro (2017) highlights the promotion of coexistence values through SL programs, which aligns with the results of the present study. Similar outcomes have been reported in contexts involving physical-motor activities (strength, endurance, range of motion) and movement-based games (Calvo Varela et al., 2019; Campos-Rius et al., 2020; Ruiz-Montero et al., 2023). Moreover, studies have shown that intergenerational programs with dependent older adults can strengthen students’ prosocial behaviors (Fu et al., 2019).

Chiva-Bartoll et al. (2020) also found that SL in primary education led to increases in specific prosocial traits such as cooperation and solidarity. Bartleet et al. (2016) reported similar findings, identifying increases in cooperative behavior through SL. These results are not surprising, as such values are central to the philosophical and pedagogical foundations of SL (Jacoby, 2015; Mitchell, 2008).

Our findings on the development of empathy are consistent with prior research emphasizing SL’s capacity to enhance students’ emotional and social commitment. In this vein, SL seems to be an effective means to promote these variables from an early age (Pérez-Ordás et al., 2021; Santos-Pastor et al., 2021). Particularly, Scott and Graham (2015) found that SL programs in primary education helped develop empathy and social responsibility. Likewise, Zorrilla (2017) reported that emotional intelligence, self-concept, and empathy improved through a game-based SL program. Vázquez et al. (2017) observed similar outcomes in another primary education SL initiative. These results are particularly important, as empathy is known to reduce antisocial behavior, anger, and verbal and physical aggression, while increasing social competence, prosocial behavior, emotional reasoning, altruism, and helping behaviors (Eisenberg et al., 2013; Robert and Strayer, 2004; Zhou et al., 2002).

Improvements in sociability observed in the intervention group are also consistent with those reported by Devlin and Warner (2017) and Scott and Graham (2015). These studies emphasized the development of solidarity and social interaction skills in the classroom. During SL experiences involving older adults, primary school students were afforded the opportunity to assume roles of responsibility, exercise decision-making, lead group activities, and navigate authentic social situations. Such engagement, in line with (Ruiz-Montero et al., 2024), could have reinforced their sense of belonging and solidarity. Similarly, Harding et al. (2010) noted that the most significant benefit experienced by students participating in SL was increased student attachment, driven by the quality of their peer interactions. Although their research focused on secondary and higher education contexts, our study offers pioneering evidence of these effects in primary education settings.

Furthermore, as shown in Table 2, the present study includes a linear regression analysis in which Model II reveals associations between Item 2 of the PE Importance Questionnaire and the total group (control and experimental), as well as a significant relationship between students’ Sociability scores and their age. In other words, the results suggest that as students grow older, those involved in SL programs become more aware of the importance of being prosocial individuals. This finding aligns with the results reported by Ruiz-Montero et al. (2022). However, it is important to note that their study was conducted with older students, so caution must be exercised when drawing comparisons, as the present sample was composed of younger primary school pupils. Given the novelty of these findings and the lack of comparable analyses in intergenerational SL interventions at the primary education level, further research is needed to explore these variables in greater depth.

In addition, gender was found to significantly influence the Leadership dimension of PB, showing a greater increase for the SL group. The development of different values in PE from a gender perspective has been reported in the past (Klomsten et al., 2005). For instance, previous studies report gender differences in tolerance and respect in PE classes, where girls obtained higher results on these dimensions (Sánchez-Oliva et al., 2012). However, we found no studies on Leadership as a specific dimension compared from a gender perspective, so these findings encourage the development of new research to analyze whether SL could specifically influence its development, especially in girls.

In conclusion, it is reasonable to consider that the characteristics of the school and its surrounding area may have influenced the results, as has been observed in other studies conducted in contexts with specific socioeconomic profiles (Farber and Bishop, 2018; Richards et al., 2013). One of the major challenges faced during the implementation of the SL intervention at the primary level was addressing various barriers that could affect its effectiveness. These included: (a) the still-developing cognitive maturity and limited autonomy of the participating students, which made it more difficult to organize and implement the process efficiently; (b) specific learning difficulties that required some students to follow individually adapted curricula in order to fully participate in the activities; (c) the necessity of training for the teachers involved in the intervention, so that activities could be aligned with both curricular objectives and the needs of the collaborating day center; (d) the complexity of assessing impact, which required the use of rubrics adapted for upper-primary students (Gómez-Rijo, 2024).

It is important to remember that this SL intervention was not solely about intergenerational education—it also represented an experiential and socially just approach to learning, particularly focused on older adults. Through observation, active experimentation, reflection, and conceptualization, participating students engaged in a full cycle of experiential learning that underscores how SL facilitated meaningful education in a real-life context involving both a school and a day care center for older adults (Kolb et al., 2014; Ruiz-Montero et al., 2020).

As a limitation, it is important to consider that this is an exploratory research and secondary variables involved could have impacted on the results. Another limitation encountered in this study was the scarcity of existing literature on intergenerational SL projects with primary school students. Several reasons may explain this gap: (a) SL has been more f institutionally supported in higher education (despite the fact that there are still many shortcomings) leaving a significant void in primary and secondary education. In fact, most of the research on; (b) a persistent belief that primary school students lack the maturity to engage meaningfully in intergenerational education through SL (González and Fuentes, 2021). Finally, it is necessary to clarify the potential non-homogeneity in age distribution between groups. As future research related to this topic, it is suggested that upcoming studies include the use of qualitative data collection techniques—such as focus groups—to enrich the findings through methodological complementarity and enhance the depth and nuance of the results.

An underlying limitation is related to developmental characteristics, such as motor coordination, which is still in progress, and the capacity for abstract thinking, which is still developing in students. According to Jiménez Vaquerizo (2019), primary and secondary school students tend to receive similar, well-organized and diverse physical education, focused on the development of their motor, interpersonal and cognitive skills.

Finally, it is important to highlight the most significant results of the present study: PE was perceived by students in the SL intervention group as an important curricular area. Most PB dimensions also showed improvements in the SL group compared to the TM intervention group.

When intergenerational experiential learning is linked to PE, it not only fosters a deeper understanding of its relevance and usefulness, but also improves prosocial behaviors in young students.

## Data Availability

The datasets presented in this study can be found in online repositories. The names of the repository/repositories and accession number(s) can be found in the article/supplementary material.
